# Establishing a web-based integrated surveillance system for early detection of infectious disease epidemic in rural China: a field experimental study

**DOI:** 10.1186/1472-6947-12-4

**Published:** 2012-02-03

**Authors:** Wei-rong Yan, Shao-fa Nie, Biao Xu, Heng-jin Dong, Lars Palm, Vinod K Diwan

**Affiliations:** 1 Division of Global Health (IHCAR), Department of Public Health Sciences, Karolinska Institutet, Nobelsvag 9, SE-17177, Stockholm, Sweden; 2 Department of Epidemiology and Biostatistics, School of Public Health, Tongji Medical College of Huazhong University of Science and Technology, Hangkong Road 13#, Wuhan, 430030, Hubei, China; 3 Department of Epidemiology, School of Public Health, Fudan University, No 138 Yi Xue Yuan Road, Shanghai, 200032, China; 4 Center for Health Policy Studies, School of Public Health, Zhejiang University School of Medicine, 866 Yuhangtang Road, Hangzhou, 310058, Zhejiang, China; 5 Future Position X (FPX), Nobelvägen 2, Box 975, SE-801 33, Gävle, Sweden

**Keywords:** Syndromic surveillance, infectious disease, early warning, resource limited settings

## Abstract

**Background:**

A crucial goal of infectious disease surveillance is the early detection of epidemics, which is essential for disease control. In China, the current surveillance system is based on confirmed case reports. In rural China, it is not practical for health units to perform laboratory tests to confirm disease and people are more likely to get 'old' and emerging infectious diseases due to poor living conditions and closer contacts with wild animals and poultry. Syndromic surveillance, which collects non-specific syndromes before diagnosis, has great advantages in promoting the early detection of epidemics and reducing the necessities of disease confirmation. It will be especially effective for surveillance in resource poor settings.

**Methods/Design:**

This is a field experimental study. The experimental tool is an innovative electronic surveillance system, combining syndromic surveillance with the existing case report surveillance in four selected counties in China. In the added syndromic surveillance, three types of data are collected including patients' major symptoms from health clinics, pharmaceutical sales from pharmacies and absenteeism information from primary school. In order to evaluate the early warning capability of the new added syndromic surveillance, the timelines and validity of the alert signals will be analyzed in comparison with the traditional case reporting system. The acceptability, feasibility and economic evaluation of the whole integrated surveillance system will be conducted in a before and after study design.

**Discussions:**

Although syndromic surveillance system has mostly been established in developed areas, there are opportunities and advantages of developing it in rural China. The project will contribute to knowledge, experience and evidence on the establishment of an integrated surveillance system, which aims to provide early warning of disease epidemics in developing countries.

## Background

In recent years, the continuous appearances of infectious diseases epidemic have attracted significant attention in China as well as in other countries over the world. In 2003, SARS firstly emerged in southern China, and then spread rapidly to the other parts in China and also in the world, resulting in the worldwide SARS epidemic. It was reported there were 5327 cases and 349 deaths in China by the end of 2003 [1]. By 5 March 2008, 30 human avian influenza cases and 20 deaths had been reported in mainland China, after the first human case was reported in 2003 [2]. In 2009 since the first imported H1N1 case was reported on May 11, the confirmed cases were consecutively reported and spread rapidly among the nation. As of Mar 31, 2010, more than 127,000 confirmed cases and 800 deaths had been reported from 31 provinces [3]. Since the beginning of 2010, an increasing number of hand, foot, and mouth disease (HFMD) cases have been reported in China. Until June 2010, there were more than 987,779 cases and 537 deaths reported. The majority of HFMD cases in China are being reported from rural communities [4].

In order to prevent and control infectious disease outbreaks effectively, the most critical step is the timely detection of epidemic premonition, which depends on effective disease surveillance systems. The sooner public health officials know about an outbreak, the more decisively they can intervene to stem its spread.

Since 2003 SARS outbreak, a web-based, daily infectious diseases case reporting system, which covered 37 notifiable infectious diseases, was instituted in April 2004 in China [5]. The current infectious disease surveillance system is majorly based on case diagnosis. In rural China, health facilities are unable to carry out laboratory tests for disease diagnosis, as they are equipped with very simple instruments. Village doctors also lack the necessary experience and up to date knowledge required for the identification and diagnosis of infectious diseases. On the other side, most people in rural areas live at lower socio-economic levels and are at a higher risk of communicable disease infection and spread, due to poor environmental and living conditions. As village health stations are unable to identify diseases, should an epidemic occur in one or more villages, it would be very difficult for the current surveillance system to detect it early enough to enable a timely response. Moreover, in rural areas people are in closer contact with wild animals and poultry, which are considered as an important risk factor for emerging disease infection. A simple, flexible and feasible early warning surveillance system for infectious disease is urgently needed in rural China.

Besides the conventional diseases surveillance system, a new surveillance system-syndromic surveillance, has become a great concern of public health policy makers [6-8]. The term "syndromic surveillance" refers to the ongoing, systemic collection, analysis, interpretation, and dissemination of data about a health-related event for use in public health action to reduce morbidity and mortality and to improve health [9,10]. As a new public health tool intended to fill the need for early warning, the syndromic approach has great advantages in promoting the early detection of epidemics and reducing the necessity of disease confirmation. It is especially effective for surveillance in resource-poor settings, where laboratory confirmation is not possible or practical, and also for detecting emerging diseases, which no additional diagnostics are requested or available. In recent years, more and more syndromic surveillance systems were established for early warning of natural diseases outbreak as well as bioterrorism events [11-13]. However they are mostly established in developed countries or areas that already have other types of advanced surveillance systems. There are no public reports of the development and implementation of a syndromic surveillance system in rural China.

In view of the urgent demand for early warning systems in rural China and of the advantages provided by syndromic surveillance, our project aims to establish an integrated surveillance system, combining a syndromic surveillance system with a case report surveillance system based on modern information technology, for the early detection of outbreaks. This project will be the first to explore if a syndromic surveillance system can complement traditional case report surveillance in rural China. The study protocol is of a European commission funded FP7 project ISSC (241900).

## Methods/Design

**Figure 1:**
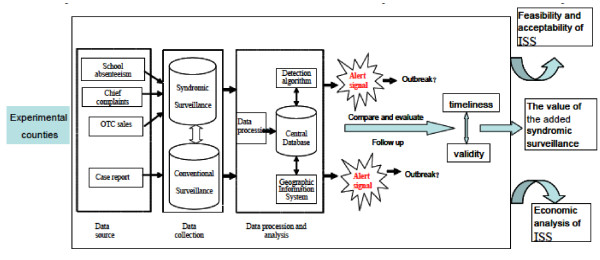
**The flow of the project**.

The project is a field experimental study. The experimental tool is an integrated surveillance system (ISS), combining an innovative syndromic surveillance with existing case report surveillance in four selected counties in China. The aberrant signals triggered by syndromic surveillance and case report surveillance will be compared and the timelines and validity of 'alerts' evaluated in order to explore the value of the new added syndromic surveillance. Then the acceptability, feasibility and economic evaluation of ISS will be further analyzed in a before and after study. The flow of the project was shown in Figure 1. Since respiratory infectious diseases, and gastrointestinal infectious diseases are the most common infectious diseases, ISS will focus on the early detection of epidemics of these two types of disease.

### Study Areas and Sample Size

Four counties in two provinces (Hubei and Jiangxi) in central China are selected as study areas. Compared with the low-income regions in western China, the areas in central China are more densely populated, thus they are more vulnerable to infectious diseases epidemics. The population characteristics, economic development, and the occurrence patterns of infectious diseases in both provinces are comparable.

In each province, two counties are purposively sampled considering the actual operational conditions for ISS. Within the selected county's jurisdiction, the townships are stratified into two levels according to their population density. At each level, 2-3 adjacent towns with similar geographical conditions are selected. More than 80% villages belonging to the selected townships are included.

### Field experimental tool-an integrated surveillance system based on modern information technology

In ISS, syndromic surveillance is being explored to complement traditional case report surveillance. Compared with conventional surveillance, syndromic surveillance is the gathering of data for public health purposes before confirmed information is available.

#### Data source in ISS

Besides the existing information source in the hospital-based case report system for notifiable diseases, three different data sources of syndrome information are collected.

(1) Patients' main symptoms when they present at health clinics: ten major symptoms are determined after literature review, experts consultation, panel discussion and field investigations, including fever, cough, sore throat, nausea/vomiting, diarrhea, rash, mucocutaneous hemorrhage, headache, convulsion and disturbance of consciousness. In addition, other basic information including patients' age, sex, home address, visiting time and date is also collected.

(2) OTC medication sales: staff in pharmacies collects the daily sold amount of the selected drugs. Different medicines related to respiratory and gastrointestinal infectious diseases are selected for surveillance based on local historical sale records. The pharmaceuticals are grouped according to Anatomical therapeutic chemical (ATC) classification system recommended by WHO as well as possible syndromes, such as antipyretics, antidiarrheal drugs, compound cold medicine, cough suppressants, etc.

(3) Primary school absenteeism: the numbers of and reasons given for absenteeism of students in primary schools are collected by teachers in charge of students' health daily. Besides the above information, the age, sex and class of each absent student are also collected.

#### Data entry and transmission

**Figure 2:**
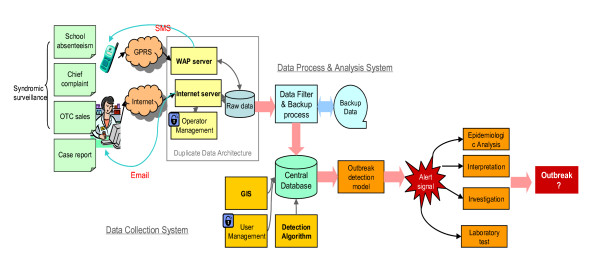
**Design flow of ISS platform**.

A web-based ISS system is developed for data collection and analysis via user-friendly interface (Figure 2). Daily data are typed or imported by data collectors in health care units, retail pharmacies and primary schools, and transferred to the central database within 24 hours mainly by Internet. If the data cannot be transferred by computer and Internet in some surveillance units, other communication tools like mobile phones, landlines, and fax machines are used for transferring instead. Staff in local CDC will receive the data and then type them into the central database manually. Raw data will be checked for logically erroneous data and duplicate records.

#### Data analysis and outbreak detection

After cleaning and backup, the data in the central database is ready for automated analyzing, which includes descriptive module and alarm trigger module. The descriptive module includes basic statistical analysis, for example the distribution of targeted symptoms across different time, space and population. The alarm trigger module incorporates models to detect aberrations from daily surveillance data. There are different methods documented in the literatures for outbreak modeling and detection research. Since there is no available historical data for modeling, models that require long-term historical data to establish the normal threshold aren't being used. Nine methods, including Moving Average (MA), Exponentially Weighted Moving Aver-age (EWMA), Cumulative Sums (CUSUM), Recursive-Least-Square (RLS) Method, Shewhart Chart (P Chart), Small Area Regression and Testing (SMART), Bayesian spatial scan statistics, Space-time Scan Statistics and What is Strange About Recent Event (WSARE) are being used in the project to detect aberrations. Additional function is developed to compare the running results of the above nine models with the same data source. If an alert is generated from more models, it indicates the possibility of being a real alarm increasing.

#### Alert reporting and dissemination

The automated analysis models run at fixed times set by users each day, and when there is an alert triggered, the system send e-mail and/or SMS alerts automatically to its specified users. The users will conduct further epidemiologic analysis, interpretation, investigation and laboratory tests in response to the alerts.

### Implementation

The implementation includes two stages. Before the implementation, all participating personnel in experimental counties will first be trained. In order to test the system and selected models in study sites, the first six months (stageI) will be used as a pilot study. The surveillance and analysis scheme will be modified in accordance with the local implementation situations. During the stageII (24 months), the daily surveillance data will be analyzed. The alert signals triggered from syndromic surveillance will be compared with those from case report surveillance for the same region and time period.

### Evaluation

The evaluation of ISS includes three aspects in comparison with the existing case report system.

#### (1) The evaluation of early warning capability

It consists of a detailed evaluation of early warning capability of the added syndromic surveillance for infectious diseases epidemics by mainly analyzing the timeliness and validity of syndrome 'alert signals' in outbreak detection compared with the corresponding case 'alert signals'. The two different kinds of time series data compared (one is syndromic surveillance time series data, the other is case report surveillance time series data as reference) are from the same time period and same region. The number, triggered time and date, sensitivity, predicted positive value of 'signals' would be compared respectively.

#### (2) Acceptability and feasibility of ISS

We will compare the acceptability and feasibility of ISS with the case report surveillance system by interviewing health workers, communities and politicians using in-depth interview and focus group discussion (FGD) tools. This will be a before and after study. The relevant communities, health workers and politicians in the village, township and county level will be interviewed before the implementation of ISS and will then be re-interviewed after one year's implementation of ISS.

#### (3) Economic evaluation of ISS

The last step of the evaluation of ISS as an early warning system will be an economic analysis that will compare ISS and the current system from a societal point of view, in terms of cost-effectiveness and cost-benefit analyses. Taking seasonal factors into consideration, the cost, effectiveness and benefit will be estimated year by year. Discounting will be taken into consideration because this project will cover 4 years. The results will be expressed in terms of incremental cost-effectiveness and incremental cost-benefit.

### Ethical issues

The study protocol has been ethically approved by institutional ethical review boards in all the participating research institutions. The rights and welfare of the participants will be protected according to the Declaration of Helsinki Ethical Principles for Medical Research Involving Human Subjects. Although personal data is collected in the project, the personal identification information does NOT appear in the final database, and all the other individual information is presented aggregately.

## Discussions

Many developed countries have used syndromic surveillance for the early detection and response to disease of public health importance, especially United States. It is reported there were more than 100 different US health jurisdictions using syndromic surveillance to augment their public health surveillance in 2003 [14]. In Asian countries, Japan, Korea and Taiwan (in China) also developed syndromic surveillance systems for diseases outbreak detection [6,15,16]. Their value in early notification of outbreaks has been confirmed in these studies [6,11-13,15-19]. However until now syndromic surveillance systems have been mostly established in developed countries or areas. Although developing settings don't have sufficient infrastructure and resources compared with developed areas, we still can find opportunities to develop syndromic surveillance in China.

1 Chinese government attaching great importance on developing an early warning surveillance system

The development of the early warning surveillance system for infectious diseases is a great concern of Chinese government and is highly relevant to national policy and to program makers in China. In 2003, state promulgated the "Regulations on Public Health Emergency" that proposed the requirements of conducting monitoring and early warning job on disease of public health importance [20]. Under the article 19 of the "Law of the People's Republic of China on the Prevention and Treatment of Infectious Diseases" amended in 2004, an early warning system for infectious diseases is required to establish in the whole country [21].

With the government's emphasis on early warning surveillance systems for infectious diseases, some pilot studies of syndromic surveillance were conducted, which provided valuable experience and evidence. In 2007, Chinese Ministry of Health (MoH) issued 'Acute infectious disease prevention and control strategies'. Chinese MoH planed to develop a comprehensive surveillance system on acute infectious diseases, including surveillance on serious clinical syndromes, unexplained death, drugs and health supplies sales, and school absenteeism, to improve the national capabilities of early warning on acute infectious diseases [22]. Thus, developing and implementing a syndromic surveillance system for infectious disease control has been put forward formally in governmental work plan.

2 Potential data sources for syndromic surveillance in rural China

An important data source for syndromic surveillance system is chief complaints, which can be obtained from outpatient logs in China. Chinese doctors are required to fill out outpatient log when each patient's visiting, which includes the patient's demographic characteristics, clinical presentations and treatment, such as name, age, gender, home address, visiting date, major symptoms, preliminary diagnosis and treatment. The log is primarily used for the management and tracking of the patients and also providing the basic information for infectious disease reporting. With spreading application of computerized hospital information system, the electronic outpatient log will become feasible in the near future.

Besides chief complaints, OTC sales information is another important type of data source for syndromic surveillance because most people experiencing symptoms would purchase OTC drugs for self-treatment before presenting at the health care facilities [23]. Recently, computer based drug sales management system is being increasingly used in retail pharmacy stores in China, even in rural areas. The electronic data of the name and quantity of medications sold per day can be fetched out from this system.

In the developing countries, infectious diseases outbreaks occurred frequently in schools or factories [24,25], so it is of great importance to collect the data of absenteeism. Absenteeism data has popularly been used to support syndromic surveillance for early detection of disease outbreaks alongside other data sources [26-28]. In primary schools in China, it is mandatory for teachers to keep records of the information of absent students and also track the prognosis of the sick students according to the regulations of Ministry of Education. Once there is a cluster of cases found, the school should report it to the local education and health bureaus timely.

3 Expanding access to modern telecommunication technology and Internet in rural China

The timely transmission of surveillance data is critical for the development of syndromic surveillance system, and the widespread application of modern telecommunication network and technology make the rapid electronic data entry, reporting and analysis possible in resource-limited areas [29].

Since the SARS epidemic in 2003, the Chinese government invested a large amount of money to built the world's largest Internet-based disease reporting system, called the China Information System for Disease Control and Prevention (CISDCP) [30]. By the end of the year 2007, the system already covered 79.04% of township hospitals, 95.99% of hospitals at or above county level and 100% of Center for Diseases Control (CDC) units in China [31]. This system has greatly improved the timeliness and completeness of infectious disease notification. Although CISDCP doesn't cover village health facilities, the usage of computer and Internet are gradually spreading in village health stations thanks to the implementation of New Cooperative Medical Scheme (NCMS), in which computer and Internet are applied to develop information management system for health insurance [32].

In remote areas without computers, mobile phones are commonly used among residents, which can be used for data reporting and transfer. For example a research conducted in 2005 revealed that adopters, potential adopters, and non-adopters of mobile phones represented 59.6%, 18.8% and 21.6% of the rural residents in Hubei province, a middle- and low- income province in China [33]. Mobile phones have already been used for infectious disease surveillance in China. In 2008, when the earthquake occurred in Sichuan which caused CISDCP paralyzed for several days, a cellular phone reporting programme was initiated emergently by China CDC for infectious disease reporting [34].

4 Early warning models and methods are being developed and tested for infectious disease surveillance in China

Automatic and periodic application of algorithm and visualization tools to detect abnormal condition timely is another important advantage of syndromic surveillance system. Exploring and developing early warning models and methods will lay valuable groundwork for the application of syndromic surveillance system in China. Since CISDCP was instituted in April 2004, China CDC has established different models to identify potential outbreaks automatically based on CISDCP and GIS technology. For example, a statistic conceptual model was established using historic surveillance data with movable percentile method by Ma JQ and his colleagues in 2007 [35]. Yin Fei, et al applied prospective space-time statistics for the early detection of measles and bacillary dysentery outbreaks with surveillance data in CISDCP [36,37]. In the study of Yang WZ and his colleagues, control chart was used to detect outbreaks/epidemics for seven infectious diseases with five-years historical data [38]. All these previous research provided experience and groundwork for the development of syndromic surveillance system in China.

In conclusion, with China's economic development, government is increasingly attaching great importance on the establishment of early warning surveillance system of disease outbreak. With the strong political support we see a window of opportunity to develop and implement a syndromic surveillance system in rural China. Currently the increasing popularity of telecommunication and GIS technology has set up an infrastructure for developing a modern electronic surveillance system. Knowledge and experience obtained from the pilot studies in China and other countries, and the theoretical research of outbreak detection models, have laid the necessary groundwork for developing a syndromic surveillance system in China.

## List of abbreviations

CDC: Center for Diseases Control; CISDCP: China Information System for Disease Control and Prevention; CUSUM: Cumulative Sums; EWMA: Exponentially Weighted Moving Aver-age; HFMD: Hand, Foot, and Mouth Disease; ISS: Integrated Surveillance System; MA: Moving Average; MoH: Ministry of Health; NCMS: New Cooperative Medical Scheme; P Chart: Shewhart Chart; RLS: Recursive-Least-Square; SMART: Small Area Regression and Testing; WSARE: What is Strange About Recent Event.

## Competing interests

The authors declare that they have no competing interests.

## Authors' contributions

WRY drafted the manuscript. WRY and VKD wrote the original protocol. SFN, BX, HJD and LP, were co-applicants on the grant application and also actively contributed to study design and to the refined protocol. WRY, SFN, BX and HJD also worked with the ethics applications at the sites. All authors read and approved the final manuscript.

## Pre-publication history

The pre-publication history for this paper can be accessed here:

http://www.biomedcentral.com/1472-6947/12/4/prepub
